# Therapeutic potential of exosomes from adipose-derived stem cells in chronic wound healing

**DOI:** 10.3389/fsurg.2022.1030288

**Published:** 2022-09-30

**Authors:** Chengmin Long, Jingru Wang, Wenjun Gan, Xinchi Qin, Ronghua Yang, Xiaodong Chen

**Affiliations:** ^1^ Guangdong Medical University, Zhanjiang, China; ^2^ Department of Burn Surgery and Skin Regeneration, the First People’s Hospital of Foshan, Foshan, China; ^3^ Key Laboratory of Regenerative Medicine, Ministry of Education, Jinan University, Guangzhou, China; ^4^ Zunyi Medical University, Zhuhai, China; ^5^ Department of Burn and Plastic Surgery, Guangzhou First People's Hospital, South China University of Technology, Guangzhou, China

**Keywords:** adipose-Derived stem cells (ADSCs), exosome (EXO), chronic wounds, wound healing, therapeutic potential

## Abstract

Chronic wound healing remains a challenging medical problem affecting society, which urgently requires anatomical and functional solutions. Adipose-derived stem cells (ADSCs), mesenchymal stem cells with self-renewal and multiple differentiation ability, play essential roles in wound healing and tissue regeneration. The exosomes from ADSCs (ADSC-EXOs) are extracellular vesicles that are essential for communication between cells. ADSC-EXOs release various bioactive molecules and subsequently restore tissue homeostasis and accelerate wound healing, by promoting various stages of wound repair, including regulating the inflammatory response, promoting wound angiogenesis, accelerating cell proliferation, and modulating wound remodeling. Compared with ADSCs, ADSC-EXOs have the advantages of avoiding ethical issues, being easily stored, and having high stability. In this review, a literature search of PubMed, Medline, and Google Scholar was performed for articles before August 1, 2022 focusing on exosomes from ADSCs, chronic wound repair, and therapeutic potential. This review aimed to provide new therapeutic strategies to help investigators explore how ADSC-EXOs regulate intercellular communication in chronic wounds.

## Background

The skin, the largest organ in humans, is a natural physical barrier against external stimulation (1). Loss of the balance between humans and the environment as a result of illness or trauma may result in substantial skin damage or even death (2, 3). Chronic wounds are long-lasting wounds that fail to achieve complete anatomical and functional repair through the normal healing process after 1 month of clinical treatment (4, 5). Chronic wounds, including vascular ulcers (venous and arterial ulcers), pressure ulcers, and diabetic foot ulcers, have complex pathogenesis and long disease courses, and are associated with high disability rates (6). Common features of chronic wounds include persistent bacterial biofilms, defective re-epithelization, decreased angiogenesis, and delayed extracellular matrix (ECM) remodeling (7, 8). Approximately 2.5% of the population in the United States is affected by chronic wounds (9, 10). According to conservative estimates, nearly $32 billion is spent on wound care, thus placing substantial pressure on the economy and healthcare system (11).

Many advanced therapies have been advocated as being effective for chronic wounds, such as negative pressure wound therapy; hyperbaric oxygen treatment; and biophysical, biological, and bioengineered therapies (4). Adipose-derived stem cells (ADSCs) are considered the most advantageous therapy for present-day regenerative medicine, given the abundant sources of adipose tissue, and the cells' outstanding proliferative ability and convenient isolation. ADSCs secrete paracrine factors and differentiate into multiple lineages (12, 13).

**Figure 1:**
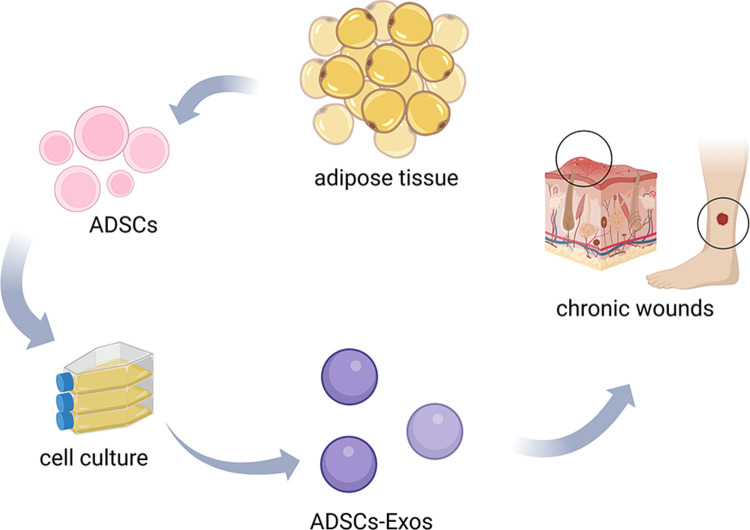
The production and applications of ADSCs-EXOs. Extracted from adipose tissue, ADSCs were collected and processed to obtain ADSCs-EXOs by cell culture. ADSCs-EXOs can apply to the treatment of chronic wound healing.

Exosomes from ADSCs (ADSC-EXOs) are small, single-membrane nanovesicles released by ADSCs through paracrine secretion, and are enriched in proteins, lipids, and nucleic acids (14–16) (Figure 1). As critical mediators of intercellular communication, they can alter the behaviors of recipient cells by transmitting signals and transporting molecules into target cells. Recent studies have shown that ADSC-EXOs had therapeutic effects in many aspects of disease, including wound healing (17, 18), organ diseases (19, 20), neurodegenerative diseases (21, 22), and cancer (23, 24). In regeneration and wound healing, ADSC-EXOs modulate persistent inflammation, angiogenesis, and ECM reconstruction. ADSC-EXO have functions resembling those of the parental stem cells, but are safer and more efficient in clinical applications, thus decreasing cell transplantation risks (25, 26).

## Adipose-derived stem cells

**Figure 2:**
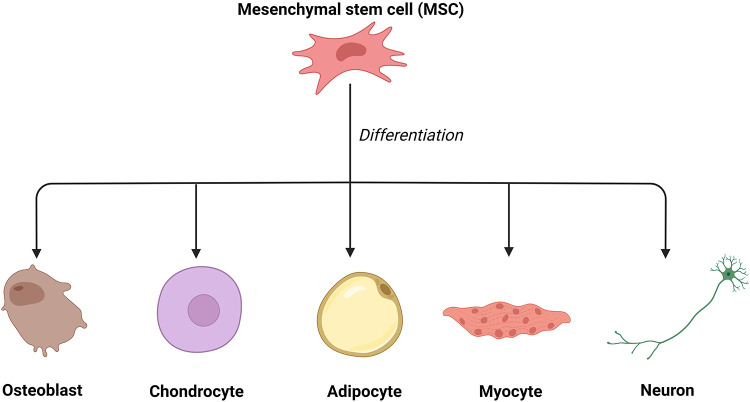
The differentiation of mesenchymal stem cells. Mesenchymal stem cells can differentiate into various mesenchymal tissue lineages, such as osteoblasts, chondrocytes, adipocytes, myoblasts, and even neurons.

Mesenchymal stem cells (MSCs) have been a promising tool in tissue engineering and regenerative medicine (27, 28) since bone marrow mesenchymal stem cells (BMMSCs) were first discovered by Alexander Friedenstein in the late 1960s (29). MSCs have been successfully applied in corneal regeneration (30), wound healing, and skin rejuvenation (12, 28, 31). In their earlier studies, Friedenstein and colleagues demonstrated that MSCs, possibly derived from the mesoderm, can differentiate into various mesenchymal tissue lineages, such as osteoblasts, chondrocytes, adipocytes, myoblasts, and even neurons (32, 33) (Figure 2). In addition, these pluripotent cells can be isolated from a variety of tissues, such as adipose tissue, muscle, blood vessels, skin, and bone marrow (34, 35).

Among all these mesenchymal stem cells, ADSCs appear to be the most advantageous for clinical applications. Compared with other types of tissues, adipose tissue is available in relatively large quantities in humans. Furthermore, many ADSCs can be isolated from adipose tissue. Prior studies have indicated that 500 times more stem cells can be harvested from adipose tissue than from equal amounts of bone marrow (36, 37). In comparison with BMMSCs, ADSCs are relatively easily obtained, owing to their subcutaneous localization. Furthermore, patients tend to feel more comfortable with less donor site morbidity (12, 38). Moreover, ADSCs have higher proliferation ability than BMMSCs (39).

In recent studies, the roles of ADSCs in wound repair treatment have been confirmed. For example, ADSCs are crucial in wound repair in diabetic foot ulcers by enhancing VEGFR3-mediated lymphangiogenesis (40). In addition, ADSCs prevent scar formation and promote wound repair in skin-deficient mice by activating the PI3K/Akt pathway (41). However, barriers to the use of ADSCs must be addressed, such as their potential oncologic properties (42). Interestingly, ADSC-EXOs, the main factors through which ADSCs exert their biological effects (43), cannot actively contribute to tumorigenesis as adipose cell-free derivatives (44).

## Characteristic of exosomes

**Figure 3:**
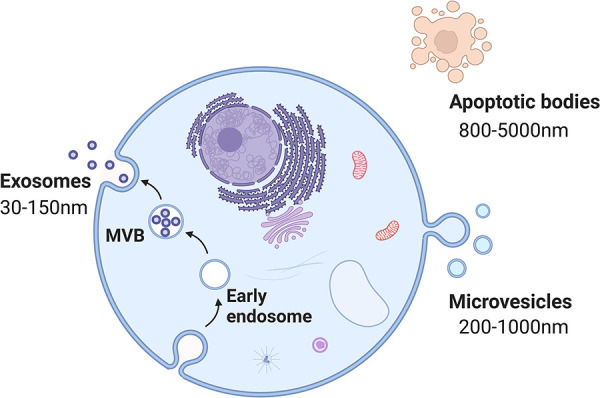
The biogenesis of each subtype of EVs. Apoptotic bodies (800–5,000 nm in size) are the result of the disintegration of apoptotic cells. Microvesicles (200–1,000 nm in size) arise from the plasma membrane. Exosomes (30–150 nm in size) originate from endosome.

Extracellular vesicles (EVs) are membrane-contained vesicles secreted by cells from multiple organisms (45). On the basis of their contents, size, and membrane composition, three primary subgroups of EVs have been defined: apoptotic bodies, microvesicles, and exosomes (46) (Figure 3). EVs generally refer to vesicles ranging from 150 to 1,000 nm released by budding from the plasma membrane (PM) (47). The term “exosomes” was initially used to describe 40–1,000-nm vesicles released by all types of cultured cells and exhibiting 5′-nucleotidase activity (48). Nevertheless, in the late 1980s, this term was used for small (30–100-nm) vesicles of endocytic origin, which are released outside cells as a result of the fusion of multivesicular bodies (MVBs) with the PM during reticulocyte differentiation (47, 49). Exosomes can be isolated from most biological fluids and cell types, such as saliva, urine, semen, nasal lavage fluid, plasma, and serum (50–53).

Exosomes contain many functional proteins, mRNAs, microRNAs (miRNAs), and complete organelles (Figure 2), which are released into the cytoplasm and mediate communication among target cells through surface membrane proteins (54, 55).

As the primary mediators of information transmission, miRNAs regulate the genes of recipient cells through self-degradation and re-expression; consequently, exosomes secreted by different cells vary in their biological functions (56). Current research on stem cell paracrine factors has indicated that exosomes secrete thousands of nutritional factors, such as stem cell factors, insulin-like growth factor I, vascular endothelial growth factor (VEGF), and transforming growth factor-β (TGF-β) (57). The uptake of exosomes by recipient cells occurs by fusion with the cell membrane, endocytosis, or receptor-ligand interaction. Adhesion-associated molecules on the surfaces of exosomes, such as glycoproteins, exosomal tetraspanin complexes, and integrins, determine the types of recipient cells (16, 58, 59).

## Inflammatory functions of ADSC-EXOs

ADSC-EXOs have immunomodulatory and anti-inflammatory effects, which remove necrotic tissue and pathogenic microorganisms from wounds, and consequently control local damage (60–62). ADSC-EXOs inhibit the differentiation and activation of T cells, thus inhibiting the release of the inflammatory factor IFN-γ and the proliferation of T cells (63, 64). ADSC-EXOs decrease adipose inflammation and obesity by regulating the phenotypic polarization of macrophages (65). Moreover, miRNAs, small, endogenous, non-coding nucleotides contained in ADSC-EXOs, play major roles in regulating metabolism and cell growth (66). One study has found that miRNA-451a enriched in ADSC-EXOs successfully promotes M1-to-M2 polarization of macrophages by downregulating macrophage migration inhibitory factor (MIF) (67). MIF is a pleiotropic pro-inflammatory mediator that participates in immune regulation *in vivo* (68). Some studies have indicated that inhibition of MIF suppresses the activation of macrophages and the expression of inflammatory factors, such as NO, tumor necrosis factor-alpha (TNF-α), and IL-6, thereby decreasing inflammatory responses and ameliorating arthritis and articular cartilage injury (68–70). Furthermore, ADSC-EXOs release many immunomodulatory proteins, such as TNF-α, macrophage colony-stimulating factor (MCSF), and retinol-binding protein 4 (RBP-4) (71). Macrophages and proteolytic enzymes are released after the destruction of cells, and subsequently digest cell debris and necrotic tissue, thus providing a suitable environment for wound repair (72–74). ADSC-EXOs have been found to contribute to wound healing in rats with diabetic foot ulcers, particularly in the presence of overexpression of nuclear factor erythroid 2-associated factor 2 (NRF2). The expression of oxidative stress-associated proteins and inflammatory cytokines is diminished (75). ADSC-EXOs have comparable properties to those of their parent cells. They can improve graft retention by upregulating early inflammation and angiogenesis (76). Similarly, ADSC-EXOs upregulate the expression of macrophage inflammatory protein-1α and monocyte chemoattractant protein-1, thus promoting early inflammation (76). Further research on immunomodulation and anti-inflammation of ADSC-EXOs in chronic wounds is needed.

## Angiogenesis regulation by ADSC-EXOs

Another function of ADSC-EXOs in wound repair is promoting angiogenesis, a dynamic process delivering sufficient nutrients and oxygen to the tissue. Emerging new capillaries, macrophages, and loose connective tissue contribute to granulation tissue formation. The elevated glucose levels in patients with diabetes can destroy the balance between vessel growth and maturation (77). In chronic wounds, perturbations in vascular integrity decrease vascularity and capillary density (78). VEGF, angiopoietin, fibroblast growth factor (FGF), and transforming growth factor (TGF) are key angiogenic cytokines in wound angiogenesis. Among these factors, VEGF-A is considered one of the most potent angiogenic factors in wounds (79). This protein, which is produced by many cells such as macrophages, binds its receptors on endothelial cells and subsequently induces migration, proliferation, and vessel growth. Mice deficient in VEGF-A 6, 7, or Flk1 8 succumb to a lack of angiogenesis early in development (80–82). ADSC-EXOs possess a higher ability to enhance angiogenesis in fat grafting by regulating VEGF/VEGF-R signaling and activating the protein kinase A (PKA) signaling pathway (83, 84). In addition to growth factors that mediate wound healing, such as VEGF-A and platelet-derived growth factor BB (PDGF-BB), ADSC-EXOs are enriched in miRNA-125a and miRNA-31 (85). ADSC-EXOs transfer miRNA-125a and miRNA-31 cargo into vascular endothelial cells (VECs), and consequently downregulate the expression of angiogenesis inhibitor Delta-like ligand 4 (DLL4), thereby promoting VEC proliferation and angiogenesis (86). Lu et al. have found that ADSC-EXOs containing miRNA-486-5p promote angiogenesis and accelerate cutaneous wound healing (87). In addition, ADSC-EXOs inhibit the overexpression of the anti-angiogenic gene hypoxia-inducible factor-1 (HIF-1) after chronic wound injury (88). We propose that ADSC-EXOs significantly affect angiogenesis, a possibility requiring further investigation.

## Proliferation and ADSC-EXOs

ADSC-EXOs have a beneficial effect in accelerating cell proliferation, which is crucial for the treatment of chronic wounds. For example, exosomes from miRNA-199-3p-modified ADSCs contribute to the proliferation and migration of endothelial tip cells (89). Moreover, ADSC-EXOs facilitate osteosarcoma progression by increasing COLGALT2 expression in osteosarcoma cells (90). In the cell proliferation stage, fibroblasts stimulate wound healing by proliferating and synthesizing large amounts of ECM components, such as collagen and elastic fibers, under the stimulation of trauma (78). ADSC-EXOs are internalized by fibroblasts and affect cell migration, proliferation, and collagen synthesis by promoting gene expression of N-cadherin, cyclin 1, proliferating cell nuclear antigen, and collagen type I and III. In a dependent manner, higher doses of exosomes can achieve faster migration rates (91). Choi et al. have found that ADSC-EXOs promote the proliferation and differentiation of dermal fibroblasts through microRNAs inhibiting genes including NPM1, PDCD4, CCL5, and NUP62, thereby contributing to the regeneration of skin fibroblasts (92). Moreover, miRNA-21 is highly expressed in adipose-derived stem cell exosomes and has been found to enhance the migration and proliferation of HaCaT cells by increasing matrix metalloproteinase-9 expression through the PI3K/AKT pathway (93).

## ECM remodeling and ADSC-EXOs

ADSC-EXOs regulate remodeling of the ECM. During the wound remodeling stage, fibroblasts differentiate into myofibroblasts, and the granulation tissue gradually becomes fibrotic; collagen gradually increases; the wound begins to contract; and scar tissue is eventually formed. ADSC-EXOs promote collagen remodeling through the synthesis of collagen types I and III in early stages of wound healing. In late stages, they inhibit collagen formation and decrease scarring (94). ADSC-EXOs prevent fibroblast-to-myofibroblast differentiation by increasing the ratio of collagen III to collagen I, as well as the ratio of TGF-β3 to TGF-β1. Moreover, ADSC-EXOs increase the expression of matrix metalloproteinase-3 (MMP3) in skin dermal fibroblasts by activating the ERK/MAPK pathway, thus resulting in a high ratio of MMP3 to tissue inhibitor of metalloprotease 1 (TIMP1), and facilitating remodeling of the ECM and diminished scarring during wound healing (92). More research on wound remodeling is needed to achieve the goals of clinical application of ADSC-EXOs.

Wound healing is a complex and dynamic physiological process that can generally be divided into four highly integrated and overlapping stages: hemostasis, inflammation, proliferation, and remodeling. These phases and their biophysiological functions must occur in an appropriate sequence at a specific time. Otherwise, interruptions, abnormalities, or prolongations in the process may result in delayed or chronic wound non-healing. ADSC-EXOs are extensively involved in the above-mentioned wound repair process through their release of various bioactive molecules (95, 96).

**Figure 4:**
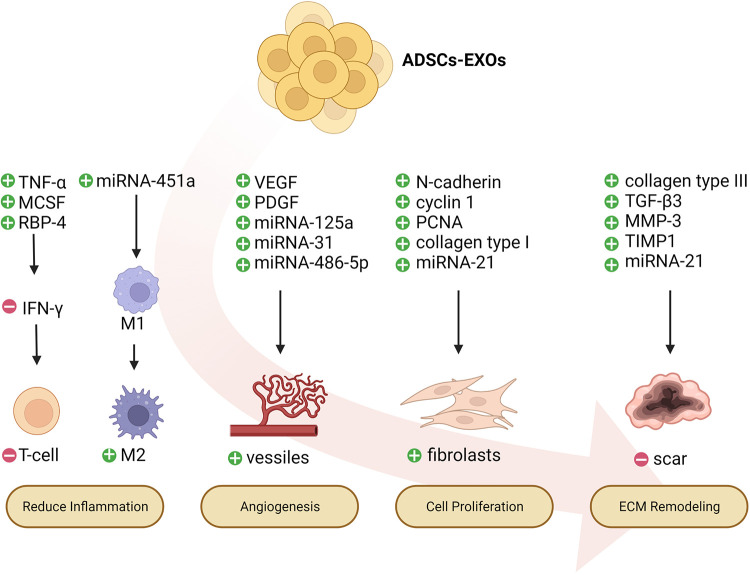
Potential mechanisms of ADSCs-EXOs regulating wound healing. ADSCs-EXOs might accelerate chronic wound repair by up-regulating early inflammation, promoting angiogenesis, enhancing proliferation, and regulating extracellular matrix (ECM) remodeling.

All types of wounds may begin as small cuts and have the potential to evolve into chronic wounds. The repair process of chronic wounds usually begins with a normal acute wound. Similar features can be found in chronic wounds, although they are classified into different categories according to etiology. Persistent infections, excessive levels of proinflammatory cytokines, as well as senescent cells that do not respond to reparative stimuli lead to chronic wounds. Definitive evidence has indicated that wound dressing along with ADSC-EXOs alleviates diabetic and infectious wound healing (97) (Figure 4).

## Challenges and prospects

The exosome field has advanced remarkably rapidly. Extensive evidence has indicated that ADSC-EXOs have robust effects on multiple stages of chronic wound repair tissue regeneration as critical mediators of intercellular communication. For example, in a diabetic mouse model of delayed wound healing, ADSC-EXOs enhance skin collagen production, angiogenesis, and cell proliferation; inhibit apoptosis; promote skin barrier function repair; and decrease inflammation in skin lesions (98). Given these properties, ADSC-EXOs may have promise in applications in chronic wound repair, skin anti-aging therapy, and scarless cutaneous repair. ADSC-EXOs show positive effects in preventing skin aging through protecting human dermal fibroblasts (HDFs) against ultraviolet B-induced photoaging damage (99, 100). Wang and colleagues have demonstrated that ADSC-EXOs promote wound repair in diabetic mice during angiogenesis and remodeling (101). In genetic therapy, ADSC-EXOs have been found to be a new therapeutic target for curing PD in patients (102).

Despite the positive results obtained in pre-clinical studies, multiple issues must be considered before clinical application of ADSC-EXOs. First, animal models for research are unable to fully reproduce the complexity of human chronic wounds, and clinical trials are scarce. Pigs or guinea pigs have skin structure somewhat similar to that in humans, thus providing a better animal model than mice in ADSC-EXO studies. Second, because they induce both effective pro-tumor and antitumor immune responses, ADSC-EXOs must be very carefully assessed in terms of safety and efficacy. Finally, technical issues such as extraction and purification of ADSC-EXOs must be simplified. The delivery and application methods are also worthy of consideration to achieve the development of large quantities of ADSC-EXOs capable of long-term storage of for clinical applications. Finally, although ADSC-EXOs appear to be a promising therapy for multiple diseases, further investigation and comprehensive information on isolating and identifying ADSC-EXOs is needed for widespread applications in clinical practice.

## Conclusion

In summary, chronic wound repair is a well-orchestrated process involving numerous factors participating in a sequence of steps. ADSC-EXOs appear to be a potential therapeutic agent for chronic wounds by promoting various stages of wound healing, including decreased oxidative stress, increased neo-vascularization, enhanced collagen deposition, and less scarring. With continuing discoveries in this field, ADSC-EXOs involved in various biological functions are expected to hold promise for treating a wide variety of disorders.
